# Semantic representation in the white matter pathway

**DOI:** 10.1371/journal.pbio.2003993

**Published:** 2018-04-06

**Authors:** Yuxing Fang, Xiaosha Wang, Suyu Zhong, Luping Song, Zaizhu Han, Gaolang Gong, Yanchao Bi

**Affiliations:** 1 National Key Laboratory of Cognitive Neuroscience and Learning and IDG/McGovern Institute for Brain Research, Beijing Normal University, Beijing, China; 2 Beijing Key Laboratory of Brain Imaging and Connectomics, Beijing Normal University, Beijing, China; 3 Rehabilitation College of Capital Medical University, China Rehabilitation Research Center, Beijing, China; Vanderbilt University, United States of America

## Abstract

Object conceptual processing has been localized to distributed cortical regions that represent specific attributes. A challenging question is how object semantic space is formed. We tested a novel framework of representing semantic space in the pattern of white matter (WM) connections by extending the representational similarity analysis (RSA) to structural lesion pattern and behavioral data in 80 brain-damaged patients. For each WM connection, a neural representational dissimilarity matrix (RDM) was computed by first building machine-learning models with the voxel-wise WM lesion patterns as features to predict naming performance of a particular item and then computing the correlation between the predicted naming score and the actual naming score of another item in the testing patients. This correlation was used to build the neural RDM based on the assumption that if the connection pattern contains certain aspects of information shared by the naming processes of these two items, models trained with one item should also predict naming accuracy of the other. Correlating the neural RDM with various cognitive RDMs revealed that neural patterns in several WM connections that connect left occipital/middle temporal regions and anterior temporal regions associated with the object semantic space. Such associations were not attributable to modality-specific attributes (shape, manipulation, color, and motion), to peripheral picture-naming processes (picture visual similarity, phonological similarity), to broad semantic categories, or to the properties of the cortical regions that they connected, which tended to represent multiple modality-specific attributes. That is, the semantic space could be represented through WM connection patterns across cortical regions representing modality-specific attributes.

## Author summary

One of the most challenging questions in cognitive neuroscience is how semantic knowledge, for example, that “scissors” and “knives” are related in meaning, can emerge from primary sensory dimensions such as visual forms. It is often assumed that in the human brain, semantics are stored in regions of the brain cortex, where distinct types of modality-specific information are transferred to and bind together. We tested an alternative hypothesis—“representation by connection”—in which higher-order semantic information could be coded by means of connection patterns between cortical regions. Combining data from behavior and brain imaging of 80 patients with brain lesions, we applied machine learning to construct the mapping models between the lesion patterns on axonal tracts (white matter) and item-specific object-naming performances. We found that specific white matter lesions produced deficits in object naming associated with the object’s semantic space, but not relevant to its primary dimension. The naming performances of semantically related objects were better predicted from white matter lesion-pattern models. That is, the higher-order semantic space could be coded in patterns of brain connections by linking cortical areas that do not necessarily contain such information.

## Introduction

One of the most challenging questions in cognitive neuroscience is how abstract knowledge emerges from more basic dimensions of information, such as visual shapes and patterns of motor action. How do we proceed from the visual shape of a pair of scissors to the knowledge that they can be used to cut things and that they are semantically related to an axe, which looks different and is manipulated differently from scissors? Research on the neural basis of semantic memory—the storage of general knowledge about the world—has revealed widely distributed brain regions supporting modality-specific attributes of objects, such as shape, color, and motion (e.g., [1,2]; see review in [3]). Nonetheless, such attribute-specific knowledge and its simple pairings are not adequate to explain the actual semantic space of objects that have quite different sensory/motor attributes but that may nonetheless be considered to be semantically similar (e.g., [4–7]). To achieve such a semantic space, various steps of binding and abstraction are assumed to occur at specific gray matter (GM) regions [6,8–11].

Although past research on semantic representation has focused on the roles of cortical regions, specific white matter (WM) tracts have been found to be necessary for semantic processing, including the left inferior fronto-occipital fasciculus (IFOF), the left uncinate fasciculus (UF), and the left anterior thalamic radiation. Damage to these tracts is associated with semantic deficits in patients [12–17]. WM is classically assumed to relay information [18–20]. In accord with this general notion, these WM tracts that are necessary for semantic processing are assumed to relay distributed information to particular GM regions (e.g., the anterior temporal lobe or angular gyrus) for binding, where concepts are represented and the “deep structures” of semantic space are formed [6,7,21]. The nature of the potential information carried by WM has never been discussed or examined.

Herein, we present results for a new notion that the WM connections, being natural binding structures, provide an alternative basis to achieve semantic representation. Distributed GM regions that represent different attribute dimensions (e.g., shape, color, manner of interaction) of the same object are connected by WM. The WM linking pattern itself would then contain multiple dimensions of information in these GM regions and, importantly, additional information about the manner of mapping among various attributes. The incorporation of these elements has been argued to be necessary for the “higher-order” semantic similarity relationships, which are not explained by attribute-specific spaces, to emerge (e.g., [7, 22]).

**Fig 1:**
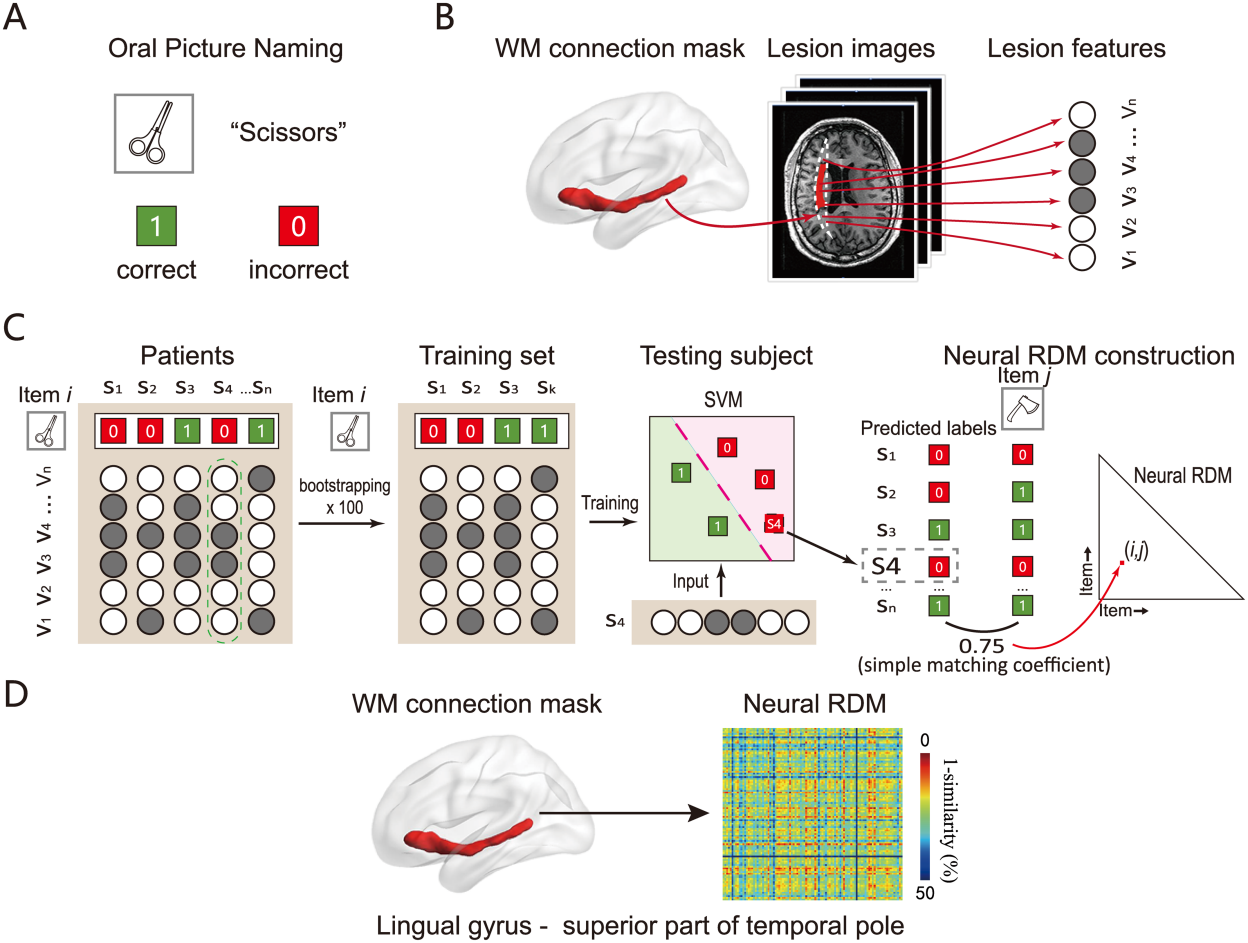
A flowchart for constructing a neural RDM in a WM connection. (A) The neuropsychological test. We asked patients to complete a picture-naming task containing 100 items. The response for each item was scored as 1 if correct or 0 if wrong. (B) The lesion mask (manually traced in T1 image) in a given patient was converted to MNI space and was then overlapped with the WM connection template constructed from a healthy population [27] to extract the voxel-wise lesion pattern on each WM connection. (C) The SVM classifier was trained on the naming accuracy of one item *i* (e.g., scissors) and lesion patterns on a WM connection in some patients (see Materials and methods) and then used to generate the predicted naming score in the testing patients (1 or 0). The correspondence (simple matching coefficient) between the predicted score and the actual naming score of each of the other items (e.g., axe) across patients was calculated. This correlation reflects to what degree the lesion features that were useful to predict naming accuracy of item *i* could also be useful to predict item *j*, and thus was taken as the neural similarity between the naming process of the training item *i* and this other item *j* (scissors–axe similarity) on this connection. All cross-item and within-item similarity could be obtained this way, resulting in a 100 × 100 similarity matrix for this connection. (D) A sample neural RDM of a WM connection (between MTG and superior ATL). The values of dissimilarity were 1-similarity (obtained in C); red indicates low dissimilarity (high similarity) and blue high dissimilarity (low similarity). The object line drawings were done by the first author Y.F.; The brain figure was generated using BrainNet Viewer [28]. ATL, anterior temporal lobe; MTG, middle temporal gyrus; RDM, representational dissimilarity matrix; SVM, support vector machine; WM, white matter.

To investigate the information coded in WM connections, we extended representational similarity analysis (RSA) [23], a highly productive method that tests the nature of representation in functional magnetic resonance imaging (fMRI) studies of cortical regions [24–26], to lesion data and WM connections. RSA examines the relationship between the representational dissimilarity matrix (RDM) derived from neural patterns and RDMs based on various types of stimulus information as a measure of information representation. The conventional neural RDM is measured by the dissimilarity of brain activity patterns induced by stimulus conditions. Here, we compute the neural RDMs with a machine-learning model using the voxel-wise lesion patterns as features to predict behavioral performance in patients with brain damage (see Fig 1). The performance in picture naming of 100 object items and the structural MRI data of 80 patients were collected. For each WM connection, a training model was built for each item (e.g., scissors) using the support vector machine (SVM) classifier with patients’ voxel-wise lesion patterns as predictive features and the naming performances of that item as labels (0, incorrect; 1, correct). The correlation between the predicted score using the classifier from that item and the actual scores of another item (e.g., axe) was taken as the neural similarity basis of these two items, based on the assumption that if this connection pattern contains certain aspects of information shared by the naming process of these two items, models trained with one item (useful features relevant for such information) should also predict naming accuracy of the other item. Once the neural RDMs are obtained from various WM connections or GM regions using this method, they can be correlated with behavioral RDMs of various object property dimensions, including the semantic RDM and four modality-specific attribute RDMs (shape, manipulation, color, and motion). Neural RDMs that are correlated with the semantic RDM even after controlling for the attribute RDMs are considered to contain “higher-order” semantic information.

## Results

### Behavioral RDMs: Semantic and modality-specific attributes

**Fig 2:**
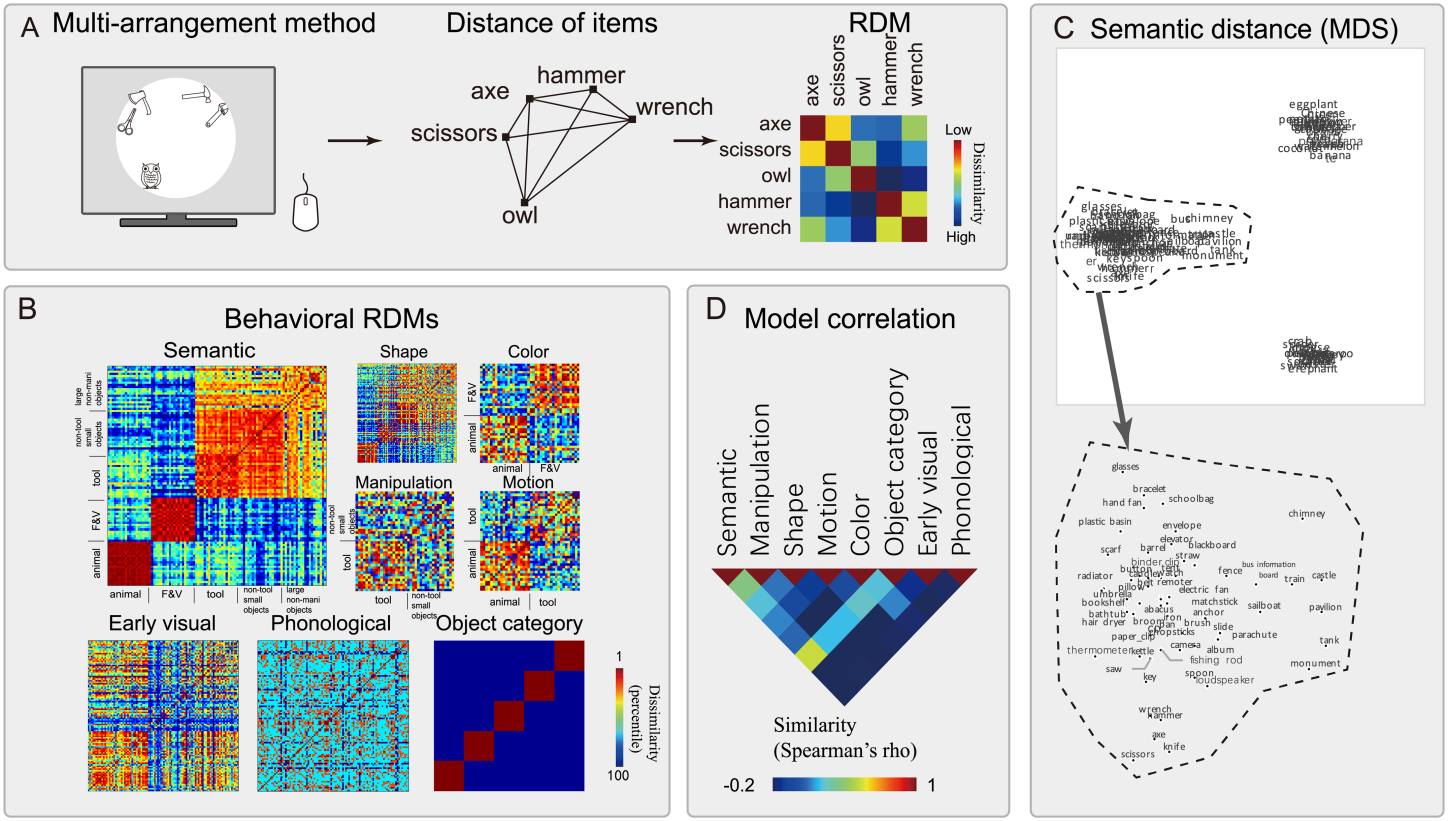
The construction and result of behavioral RDMs. (A) The multi-arrangement method. Twenty college students were asked to arrange object pictures according to their semantic (or modality-specific attribute) relatedness by dragging the items on a screen with a mouse. The distances between items on the screen would transform into an RDM. If two items, e.g., scissors and axe, showed a close distance, then they were assigned a low value in the RDM. (B) The results of the behavioral RDMs. Three broad types of distances were measured: semantic similarity, modality-specific attributes (shape, manipulation, color, and motion), and control models that are also potentially relevant to object naming (early visual, phonological, and object category matrix). The values of dissimilarity were transformed to percentile for display. Red indicates low dissimilarity (high similarity) and blue high dissimilarity (low similarity). (C) Visualization of the semantic RDM using multidimensional scaling. (D) The correlations among various behavioral RDMs. The object line drawings were done by the first author Y.F. The underlying data for this figure can be found at https://osf.io/h7upk/?view_only=52b8f86cffa14ed4844e4a1b9cd429cb. F&V, fruit and vegetable; MDS, multidimensional scaling; RDM, representational dissimilarity matrix.

Behavioral RDMs for the semantic, shape, manipulation, color, and motion features of 100 objects (20 animals, 20 fruits and vegetables, 20 tools, 20 non-tool small objects, and 20 large non-manipulable objects) were generated using a multi-arrangement method [29]. In this task, 20 college students were instructed to arrange the items by a particular dimension of interest on a computer screen, and the distance among items was derived, resulting in an RDM (see Fig 2A).

The semantic RDM was visually clustered into three domains: animals, fruits and vegetables, and man-made objects (tools, small non-tool objects, large non-manipulable objects; see Fig 2B & 2C). Visualization of the semantic RDM using multidimensional scaling (Fig 2C) further revealed that within each category, words with closer semantics tended to share similar function (e.g., scissors and knife), share certain distinct features, or belong to finer subordinate categories (e.g., peanut and potato). The semantic RDM and the four modality-specific attribute RDMs were intercorrelated to various degrees (Fig 2D; semantic with shape: *r* = 0.35; with manipulation: *r* = 0.47; with color: *r* = 0.23; with motion: *r* = 0.27; *p* < 10^−9^).

### WM neural RDMs: Lesion-naming model decoding

Neural RDMs were generated for each of the 688 WM connections (S1A Fig) that were identified through deterministic tractography across 90 automated anatomical labeling (AAL) regions based on the diffusion tensor imaging (DTI) data of 48 healthy controls [27]. To generate the neural RDM for each WM connection, we performed lesion-naming model decoding using voxel-wise lesion patterns and item-level naming responses. For 80 patients with brain damage, lesion patterns in each WM connection (with each voxel in the WM connections labeled as “lesion” or “intact”) for each patient were obtained by overlapping the manually traced lesion mask (converted to the MNI) space) with the WM mask (see Fig 1). A total of 680 out of 688 WM connections with adequate lesion coverage (see Materials and methods; see also S1E Fig for the lesion distribution map) were included in the following analyses. The patients’ naming performances for each of the 100 pictures were collected (performance distribution in S1B Fig).

WM neural RDMs were generated using item-based lesion-naming prediction models. For 197 connections, the lesion-naming models had successful within-item prediction averaged across all items (Bonferroni *p* < 0.05; diagonal in Fig 1D). That is, they yielded successful naming prediction models and were the connections that we considered in the following analyses. Of these connections, 185 were located in the left hemisphere and 12 in the right hemisphere (S1C Fig). For each of these WM connections, we computed the correspondence between the predicted scores using SVM classifiers built using the training patients’ lesion patterns and the naming scores of one item and the actual naming score of another item in the testing samples across testing iterations. This between-item correlation was taken as the similarity value for this item pair in the neural RDM, based on the assumption that if this connection pattern contains certain aspects of information shared by the naming process of these two items being captured by the SVM model, models trained with one item should also predict naming accuracy of the other item. Worth clarifying is that this procedure does not depend fully on the correlation between the actual naming accuracies across item pairs but also to what degree the potentially shared underlying properties for their naming process are supported by each WM connection (as captured by the SVM models). For example, for connections supporting phonological processing, the SVM models may pick up phonological properties and result in higher correlation between phonologically related pairs; those supporting semantic processing may pick up semantic properties and result in correlation between semantically related pairs. The resulting 100 × 100 (-item) lesion-naming prediction similarity matrix was transformed to be the neural RDM of this connection (1-prediction similarity, Fig 1D).

### RSA results: Semantic representation in WM connections

Using RSA, the correlations between the WM neural RDMs and the semantic RDM were assessed. Significantly positive correlations were obtained in 60 WM connections (*r* = 0.03–0.11, false discovery rate [FDR] *q* < 0.05; see S1D Fig). These WM connections connected widely distributed regions across the left hemisphere, and approximately half (31/60) of the connections had at least one of the connected nodes located in the temporal lobe. The most densely connected regions (degree z-score > 1) were the middle temporal gyrus (MTG), superior temporal gyrus (STG), orbital part of middle frontal gyrus, inferior parietal lobule (IPL), and precentral gyrus.

**Fig 3:**
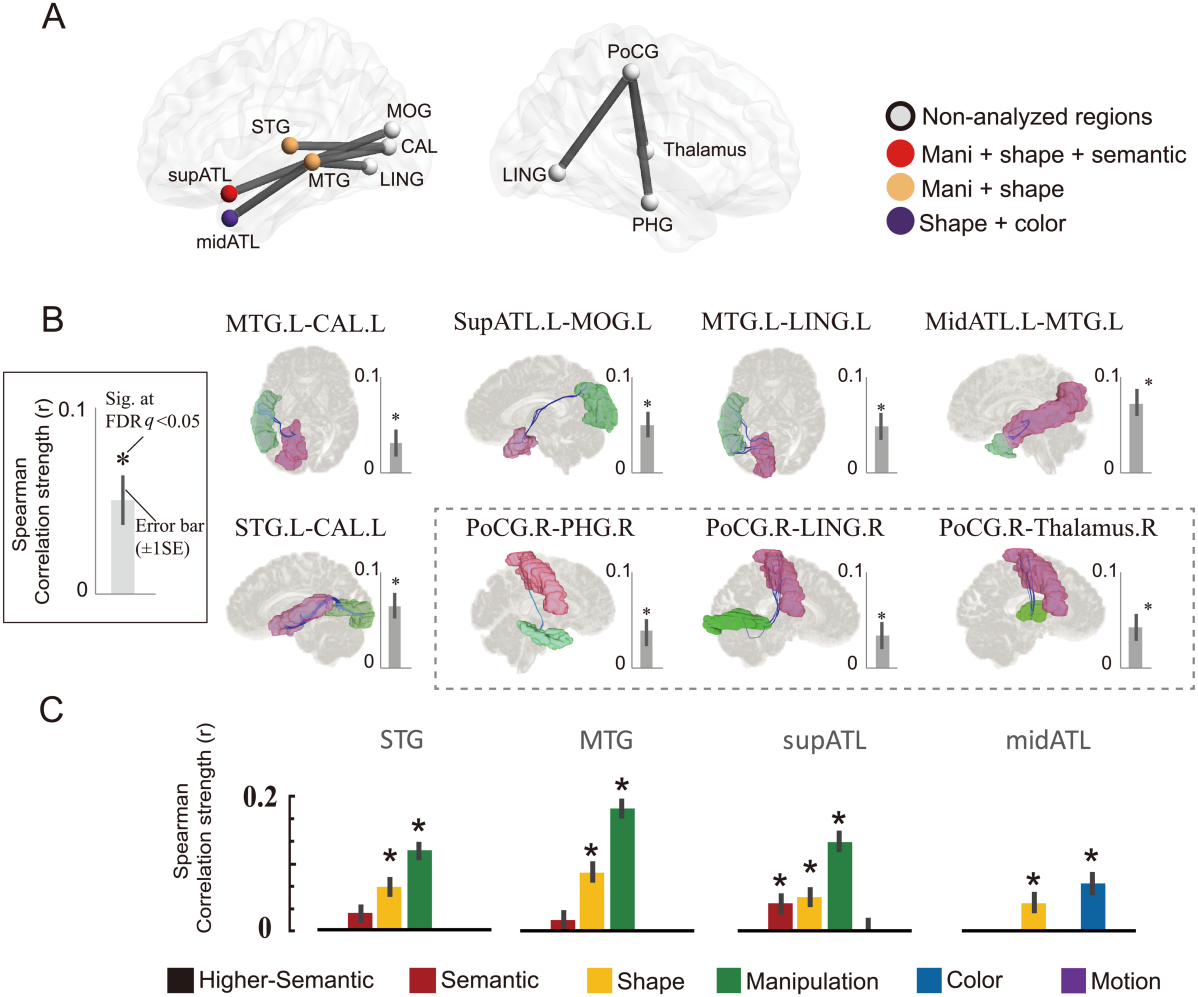
WM connections representing higher-order semantic space. (A) Eight WM connections representing higher-order semantic space, with 11 GM regions being connected. The regions that fail to show successful within-item prediction or in right hemisphere are rendered gray. The four colored regions represent raw semantic effects or modality-specific attributes (red for manipulation, shape, and semantic; orange for manipulation and shape; and purple for shape and color). (B) The WM connections reconstructed using the HCP database. The blue streamlines are the WM connections between two GM regions (rendered in red and green). The masks of WM connections reconstructed with current data are shown in S2 Fig. The RSA results of the eight WM connections, with bars showing the correlation strength (*r* value) between neural and semantic RDMs and error bars indicating ±1 standard error based on 1,000 times bootstrap resampling (see [23] for details) of the neural and behavioral RDM sets. The three WM connections did not survive all validation tests were shown in the dashed box. (C) The GM nodes representing semantic and modality-specific knowledge. The bar figure shows the RSA correlation strength (*r* value) of the semantic and modality-specific attributes in the colored GM regions in (A); the error bars indicate ±1 standard error; only positive values are shown. Note that for the superior ATL, in which RSA with semantic space was significant, its semantic effects diminished when controlling for modality-specific attribute RDMs. Asterisks indicate FDR *q* < 0.05. The object line drawings were done by first author Y.F.; the brain illustrations were generated using BrainNet Viewer [28] and DSI Studio (http://dsi-studio.labsolver.org/). The underlying data can be found at https://osf.io/h7upk/?view_only=52b8f86cffa14ed4844e4a1b9cd429cb. ATL, anterior temporal lobe; CAL, calcarine sulcus; FDR, false discovery rate; GM, gray matter; HCP, Human Connectome Project; LING, lingual gyrus; midATL, middle anterior temporal lobe; MOG, middle occipital gyrus; MTG, middle temporal gyrus; PHG, parahippocampal gyrus; PoCG, postcentral gyrus; RDM, representational dissimilarity matrix; RSA, representational similarity analysis; STG, superior temporal gyrus; supATL, superior anterior temporal lobe; WM, white matter.

What about semantic effects that could not be explained by modality-specific attributes, peripheral factors, or broad semantic categorical effects? We controlled for the effects of all four modality-specific attributes, two peripheral variables (the early visual and phonological) and semantic category matrix (labeling within-category pairs 1 and between-category pair 0) using partial correlation. The semantic effect was consistently significant in eight WM connections (*r* = 0.03–0.07, FDR *q* < 0.05; Fig 3A–3C). Table 1 presents the detailed statistical results before and after, including these variables as covariates. These eight connections were considered to represent (relatively) higher-order semantic space. Five of them were located in the left ventral visual pathway and connected occipital regions (middle occipital gyrus, calcarine sulcus, and lingual gyrus) and temporal regions (STG, MTG, superior anterior temporal lobe [ATL], and middle ATL). The three remaining WM connections were located in the right hemisphere, connecting the postcentral gyrus with the thalamus, lingual gyrus, and parahippocampal gyrus. These reconstructed connections are shown in Fig 3B and S2 Fig.

**Table 1:** The RSA results of the WM connections showing significant effects of higher-order semantic space. For each connection, results (r(*p*)) are shown for the higher-order semantic space, raw semantic space, and broad object category, before or after controlling for various types of stimulus properties and in various subsets of patients. R values are the Spearman *r* between the neural RDM in the corresponding connection and the semantic RDM with various other properties controlled for.

Analysis aspect	Control variables	WM connections							
		MTG_L—CAL_L	MTG_L—LING_L	MidATL_L—MTG_L	SupATL_L—MOG_L	STG_L—CAL_L	PoCG_R -LING_R	PoCG_R—PHG_R	Thalamus_R—PoCG_R
Higher-order semantic RSA									
	(control for modality-specific attributes^a^ and the other relevant matrices^#^)								
		0.032(0.024*)	0.049(<0.001*)	0.073(<1 × 10^−6^*)	0.051(<0.001*)	0.065(<0.001*)	0.04(0.005*)	0.034(0.017*)	0.043(0.002*)
	(control for modality-specific attributes^b^ and the other relevant matrices^#^)								
		0.021(0.147)	0.029(0.045)	0.065(<0.001*)	0.044(0.002*)	0.05(<0.001*)	0.036(0.012*)	0.032(0.026)	0.036(0.011*)
Higher-order semantic RSA: Effect of modality-specific attributes									
	(control for modality-specific attributes^a^)								
		0.045(0.001*)	0.066(<0.001*)	0.065(<0.001*)	0.046(0.001*)	0.065(<0.001*)	0.067(<0.001*)	0.052(<0.001*)	0.059(<0.001*)
Higher-order semantic RSA: Types of lesion									
	(using only stroke patients, control for modality-specific attributes^a^ and the other relevant matrices^#^)								
		0.056(<0.001*)	0.081(<1 × 10^−6^*)	0.077(<1 × 10^−6^*)	0.067(<0.001*)	0.085(<1 × 10^−6^*)	0.017(0.245)	0.017(0.234)	−0.004
	(using only patients with unilateral left hemispheric lesions, control for modality-specific attributes^a^ and the other relevant matrices^#^)								
		0.031(0.028)	0.049(<0.001*)	0.039(0.007*)	0.049(<0.001*)	0.05(<0.001*)	-	-	-
Higher-order semantic RSA: Effect of the GM nodes									
	(control for modality-specific attributes^a^ and the other relevant matrices^#^ and the neural RDMs of two GM nodes being connected)								
		0.038(0.007*)	0.057(<0.001*)	0.096(<1 × 10^−6^*)	0.058(<0.001*)	0.062(<0.001*)	0.026(0.063)	0.005(0.723)	−0.038
Raw semantic RSA									
	(control for the other relevant matrices^#^ and not modality-specific attributes)								
		0.049(<0.001*)	0.06(<0.001*)	0.111(<1 × 10^−6^*)	0.085(<1 × 10^−6^*)	0.089(<1 × 10^−6^*)	0.019(0.193)	0.035(0.013*)	0.028(0.045)
Object category RSA									
	(control for the low-level visual and phonological matrices)								
		0.016(0.25)	0.017(0.243)	0.014(0.323)	0.011(0.431)	0.005(0.739)	0.001(0.943)	0.005(0.747)	−0.001

*Positive correlation values that survived FDR correction (*q* < 0.05). When controlling for the modality-specific attributes, two ways (a and b, below) of dealing with items not having certain modality ratings were both considered.

^a^Setting those cells as being most dissimilar, “1.”

^b^Setting them as missing values, “NaN.”

^#^Low-level visual, phonological, category.

Abbreviations: CAL, calcarine sulcus; FDR, false discovery rate; GM, gray matter; L, left; LING, lingual gyrus; MidATL, middle anterior temporal lobe; MOG, middle occipital gyrus; MTG, middle temporal gyrus; NaN, not a number; PHG, parahippocampal gyrus; PoCG, postcentral gyrus; R, right; RDM, representational dissimilarity matrix; RSA, representational similarity analysis; STG, superior temporal gyrus; SupATL, superior anterior temporal lobe.

To examine the degree to which the semantic effects we observed on these WM connections reflect effects of broad semantic category, we also checked the RSA effect of the category matrix (correlating the neural RDM and the category RDM) and found that none of these connections had significant effects of the semantic category (*p* > 0.05, Table 1).

### Validation analyses

To consolidate the main results above, we further performed validation analyses to test the following concerns: (1) The WM mask we adopted was constructed using DTI data acquired on a scanner with a low magnetic field (1.5 T) and 32 directions. Was the WM connection construction accurate and unaffected by crossing-fiber problems? (2) To maximize power, we included patients with multiple etiologies (84% stroke and 16% traumatic brain injury [TBI]) and lesion distributions (37.5% lesion in the left hemisphere only, 43.8% lesion in bilateral hemispheres, and 18.8% in the right hemisphere only). Were the results systematically affected by disease type or hemispheric differences?

#### The quality of the WM fiber tracking

We reconstructed WM connections using diffusion data from a public state-of-the-art connectome database—the Human Connectome Project (HCP)—to ensure that the WM connections we adopted [27] were not false connections. The HCP data were acquired with a high angular resolution diffusion imaging (HARDI) sequence and therefore allowed for complex diffusion models to handle the cross-fiber issues. For the eight WM connections with higher-order semantic representation that we observed in the main results above, the WM connections reconstructed from the HCP data and our DTI data were visually very similar when projected onto the 3D brain (see S2 Fig).

#### Controlling for the effects of patient disease type and lesion hemisphere

There was no systematic difference in the naming scores between stroke and TBI patients (*t*_*78*_ = −1.30, *p* = 0.20). We computed the neural RDMs in WM connections using only data from the 67 stroke patients, and the RSA results across the five left higher-order semantic WM connections remained highly consistent with those in the results using all patients, but the effects in right hemisphere diminished (*r* = −0.004–0.017, FDR *q* > 0.05). Using only patients with unilateral left hemispheric lesions (30 patients), we also obtained results that were generally consistent with the main results: all but one higher-order semantic WM connections retained significance (FDR *q* < 0.05, except for the one connecting the MTG and calcarine sulcus, *r* = 0.03, uncorrected *p* < 0.05, see Table 1).

### RSA results: The representation content of GM nodes that are connected by semantic WM connections

What types of representations are linked by the WM connections that represent the semantic space? Do the WM connections simply relay semantic information that has already been encoded in the GM nodes, or do they contain information that cannot be accounted for by representation in the GM nodes? We tested the representational contents of the seven GM nodes that were connected by the five higher-order semantic WM connections whose effects remained robust in the validation tests (see Table 1). Four GM regions had successful within-item naming prediction and were considered in the RSA analysis: superior ATL, middle ATL, MTG, and STG. The neural RDM for each GM node was constructed using the same method as with the neural RDMs of the WM connections.

We found that the higher-order semantic representation in the five semantic WM connections cannot be simply explained by GM information (Fig 3D; S1 Table): when correlating the GM neural RDMs with the semantic RDM (controlling for peripheral and categorical matrices), only the superior ATL reached significance (*r* = 0.04, FDR *q* < 0.05). However, this effect could be explained by modality-specific attribute representations. After controlling for the four modality-specific attribute matrices, none of the four GM nodes significantly correlated with the semantic RDM at either the conventional threshold (FDR *q* < 0.05) or a less stringent threshold (uncorrected *p* < 0.05, see S1 Table). Additionally, when testing the higher-order semantic representation in the five WM connections by further adding the neural RDMs of the two GM nodes being connected as additional confounding variables, the results remained unchanged (see Table 1). We further constrained our WM connection mask with a WM mask constructed by T1 segmentation (conducted using SPM8 in MNI T1 template, default parameters) to offer a clear WM boundary, i.e., containing only WM voxels. We then recomputed the higher-order semantic RSA in these WM connections using only the voxels within the WM mask and found that the effects in all five WM connections remained significant (FDR *q* < 0.05, *r* = 0.03–0.07, SD = 0.01).

If not semantic, do these GM nodes code modality-specific attributes? We correlated the neural RDM of each GM node with each of the four modality-specific attribute RDMs (shape, manipulation, color, and motion; Fig 3D & S1 Table; the three control matrices—low-level visual, phonological, category—were controlled for). The superior ATL, MTG, and STG were significantly correlated with the shape and manipulation RDMs (shape: *r* = 0.04–0.08, manipulation: *r* = 0.12–0.16, FDR *q* < 0.05). The middle ATL was significantly correlated with the shape and color RDMs (shape: *r* = 0.04, color: *r* = 0.06, FDR *q* < 0.05).

Finally, we conducted a whole-brain analysis across all 90 AAL GM nodes. In addition to superior ATL, the neural RDMs of the left IPL, precentral gyrus, and postcentral gyrus were significantly correlated with the semantic RDM (*r* = 0.04–0.05, FDR *q* < 0.05), but none of these or any other GM regions retained significance after controlling for the four modality-specific attribute matrices (FDR *q* < 0.05).

## Discussion

To test the potential WM basis of semantic representation, we developed a structural-property-pattern-based RSA approach by applying machine learning to lesion and behavioral data in patients to derive item-based neural RDMs for WM connections. We found that a set of WM connections connecting occipital/middle temporal regions and anterior temporal regions represented a semantic space that was not explained by broad semantic categories or the effects of modality-specific attributes and, hence, was addressed as higher-order semantic representation. Such semantic effects were not fully explained by the properties of the GM nodes that were connected. Although the neural RDM of a connecting node—the superior ATL—correlated with the semantic RDM, such effect diminished after controlling for modality-specific attributes. Instead, these GM nodes tended to represent modality-specific attributes, including shape and manipulation in the superior ATL, MTG, and STG and shape and color in the middle ATL.

First, it should be noted that we inferred semantic effects to be higher-order when they were not explained by linear combinations of the classical modality-specific attributes for objects. The potential effects of some untested modalities or certain nonlinear combinations across various modalities could not be fully excluded. Also, subjectively judged semantic distance might be a rather composite measure that is driven by multiple semantic dimensions, which may have different neural bases (e.g., [30]). Under the current (conventional) operation, these WM connections that represent higher-order semantics tend to lie in several major pathways that have been associated with semantic processing using univariate lesion-behavior correlation or intraoperative stimulation [12,16,21,27,31]. These connections partly belong to IFOF, and the inferior longitudinal fasciculus (ILF) (the overlapped voxels with the Johns Hopkins University WM template: IFOF [32%], ILF [71%], and minimally on the minor forceps [6%] and superior longitudinal fasciculus [8%]). Lesion or atrophy in IFOF is associated with semantic deficit severity in patients with stroke and in patients with semantic dementia [12,27,32]. A similar result was also found with ILF in semantic dementia [16,33]. Additionally, direct intraoperative stimulation of IFOF induces semantic errors [34,35]. Our current findings based on multivariate RSA demonstrate that the organization of specific connections among these large WM tract bundles represent the fine-grained semantic space. Items closer in semantic space are represented by more similar WM patterns in these specific connections. Note that it is well known that patients’ specific naming errors may vary from session to session [36]. The WM lesion pattern observed here is likely associated with some aspects of semantic space rather than with specific items. The damage of such specific aspects of semantic space would result in noisy/impaired representation for a range of items sharing that space, resulting in potentially different outputs at different time points. Such semantic space was nonetheless much finer than broad semantic categories, however, as the RSA results were robust after controlling for the categorical matrix. It is also well known that patients may make different types of errors, such as phonological and semantic paraphasias, which may be originated from different cognitive stages. Our approach here pulled all types of naming errors together, and the RSA results of correlating the neural RDM with different RDMs (semantic versus phonological/visual) presumably reflect the neural basis of different error types, which should be directly examined in future research.

What is the relationship between the WM representations and the nature of the GM regions that they connect? First, we indeed observed that one of the seven linked GM regions was related to semantic space—superior ATL. The finding that lesion-pattern-behavior (neural) RDM in the superior ATL correlated with semantic space before regressing out the effects of modality-specific attributes converges nicely with the accumulated evidence about the cortical representation of semantics from fMRI and neuropsychological studies. ATL is the region with the strongest atrophy in patients with semantic dementia, which is marked by semantic deficits [6,7,31,37,38] and is sensitive to multiple modalities of object attributes [39,40]. Unlike the WM connections related to higher-order semantic space, the semantic effect in the superior ATL could be explained by the effects of modality-specific attributes. Worth noting is that ventral ATL was not scrutinized because it was not a node in the AAL parcellation we used but was included in the fusiform and inferior temporal nodes. What should be highlighted, however, is that the positive effects of higher-order semantic representation in the WM connections are significant and are not simply inheriting the properties of the connected GM nodes. Several higher-order semantic WM connections observed here connected ATL with other regions, inviting further questions about whether it is the integrity of ATL or of the ATL-related WM connections that make stronger contributions to the semantic deficits in semantic dementia patients.

While our results certainly do not argue against the possibility that there are specific GM regions supporting semantic representation, we found that the GM nodes being connected by the WM connections obtained here tended to represent multiple modality-specific object properties. Of the four GM regions we could test, the MTG, STG, and superior ATL represented shape and manipulation properties, and the middle ATL represented shape and color properties. These results converge nicely with the fMRI literature studying the sensitivity of these regions for object attributes. For instance, the effects of various attributes were recently tested using parametric modulation analyses [2], which found that the posterior MTG was sensitive to both shape and manipulation knowledge. Coutanche and Thompson-Schill [39] found that the ATL codes the integration of color and shape, and Peelen and Caramazza [40] found that the ATL codes both manipulation and location. The STG was sensitive to motion properties in Fernandino et al. [2] but not in our study, perhaps due to different parcellation scales regarding the finer structure within this region. Note that many studies about the attribute-specific property representations have revealed results in sensory and motor cortices (e.g., shape in the lateral occipital/temporal cortex: [26,41]; color in the ventromedial occipital cortex such as lingual gyrus: [42–44]). However, these regions could not be tested in our data given their chance-level lesion-naming prediction performance, which could either be due to low lesion distribution in these regions (see S1E Fig for lesion distributions) or because the specific dimensions they represent are unnecessary for object picture-naming behavior. It may also be the case that higher-order semantic space is formed by binding multiple, rather than single, pairs of attributes. Consistent with this speculation, it has been shown that computation simulation models with a convergent architecture, in which intermediate units code multiple types of dimension pairings, were better at capturing the “deep” structure of conceptual space and promoting generalizations across semantically related items that were not apparently similar along single dimensions [22].

What is the mechanism of coding higher-order semantic information in WM that connects multiple modality-specific attributes? One potential mechanism could be through synchronized firing of specific sensory and motor patterns for objects. Consider when people use a pair of scissors: the neurons that represent the attributes across various modalities—e.g., shape, haptics, ways of grasping and manipulating it, seeing the consequence of using it (things being cut)—fire together. Such functional co-activation across a wide range of attributes occurs often when we see or use scissors, which enhances the structural connection between neurons within and across dimensions of the same object. WM provides a basis for such synchronization between distant cortical regions [45]. These synchronizations also lead to the building and tuning of WM connections, because neuronal activity traveling through axons can affect the properties of myelin sheaths in the active circuit; for example, electrical activity in the axon induces myelination [46,47]. This interactive process results in the WM basis of a multidimensional representation of “scissors,” which is closer in the higher-order semantic space to concepts such as “axe” or “paper.” The formation and modulation of the WM microstructure underlying these representations can be affected by our experiences, which is the basis of acquiring new concepts and of the coloring of existing concepts. Ample evidence describes how WM is affected by experience. Early-life experiential deprivation in animals and humans leads to decreased myelin sheath thickness and WM volume [48,49], whereas these parameters increase when the organism is placed in a rich experiential environment [50]. Reading training [51] and music practice [52,53] during childhood lead to increased fractional anisotropy in WM. The acquisition of motor skills changes the WM microstructure [54,55]. The exact relationship between WM microstructure and the functional coupling between cortical regions for various representational dimensions warrants further studies.

A final methodological note is that the approach we developed here—building neural RDMs using machine learning with structural lesion data and condition-specific performances—could be easily adapted to other cognitive issues and all kinds of brain structural integrity measurements, including DTI indices (e.g., fractional anisotropy, mean diffusivity) or voxel-based morphometry measures for both patient and healthy populations. For the current study, we chose to focus on manually traced lesion on the T1 image (with reference to T2) because it captures the structural damage in our specific patient group (mostly stroke) in a most straightforward fashion. RSA, an approach that connects major branches of systems neuroscience—brain-activity measurement, behavioral measurement, and computational modeling [23]—could now be extended to an additional branch, i.e., brain structural measurement.

In conclusion, using a structural-property-pattern-based RSA approach, we found that the WM structures mainly connecting occipital/middle temporal regions and anterior temporal regions represent fine-grained higher-order semantic information. Such semantic relatedness effects were not attributable to modality-specific attributes (shape, manipulation, color, and motion) or to the representation contents of the cortical regions that they connected and were above and beyond the broad categorical distinctions. By connecting multiple modality-specific attributes, higher-order semantic space can be formed through patterns of these connections.

## Materials and methods

### Participants

Eighty patients with brain damage participated in the present study. The patient group (60 males, 20 females) was recruited from the China Rehabilitation Research Center with at least 1 month post-onset (mean = 6.09; SD = 11.69; range: 1–86 months) and premorbidly right-handed. The majority suffered from stroke (*n* = 67) and others suffered from TBI (*n* = 13). The patients’ mean age was 45 years (SD = 13; range: 19–76 years) and mean years of formal education was 13 (SD = 3; range: 2–19). Twenty additional college students (10 males; mean age = 22.9, SD = 2.45, range = 19–27) participated in the multi-arrangement experiment for the behavioral RDMs. This study was approved by the Institutional Review Board of the State Key Laboratory of Cognitive Neuroscience and Learning, Beijing Normal University (IORG0004944), adhering to the Declaration of Helsinki for research involving human subjects. All participants gave informed written consent.

### MRI data collection and preprocessing

Each subject was scanned using a 1.5T GE SIGNA EXCITE scanner with an 8-channel split head coil at the China Rehabilitation Research Center. We collected two types of images: (1) high-resolution 3D T1-weighted MPRAGE images in the sagittal plane with a matrix size = 512 × 512, voxel size = 0.49 × 0.49 × 0.70 mm^3^, repetition time (TR) = 12.26 ms, echo time (TE) = 4.2 ms, inversion time = 400 ms, field of view (FOV) = 250 × 250 mm^2^, flip angle = 15°, and slice number = 248; and (2) FLAIR T2-weighted images in the axial plane with a matrix size = 512 × 512, voxel size = 0.49 × 0.49 × 5 mm^3^, TR = 8,002 ms, TE = 127.57 ms, inversion time = 2 s, FOV = 250 × 250 mm^2^, flip angle = 90°, and slice number = 28. To improve the image quality, the T1 image was scanned twice. The two scans were then co-registered and averaged for the following analyses. All imaging data can be found at the Open Science Framework database (URL: https://osf.io/h7upk/?view_only=52b8f86cffa14ed4844e4a1b9cd429cb).

### Materials, neuropsychological testing, and behavioral RDM construction

#### Materials

One hundred colored photographs of objects, with an equal number of items from five semantic categories (animals, fruits and vegetables, tools, small non-tool artifacts, and large non-manipulable objects), were used in the neuropsychological testing and behavioral RDM construction.

#### Neuropsychological testing

Patients underwent an oral picture-naming test outside the scanner. They were asked to name each object on a computer screen. The first complete response was scored. Responses were scored as 1 if correct or 0 if wrong.

#### Behavioral RDM construction

The semantic RDM was based on a multi-arrangement method [29]. Each subject judged the semantic distance among 100 objects in the oral picture-naming task by arranging them on a computer screen. The distance between any two objects on the screen reflected their semantic distance. The subjects were instructed to “arrange objects according to how similar they are in meaning; for instance, the meaning of ‘rock–cell phone’ has little in common so they should be dragged far apart; ‘rock–sand’ has high similarity in meaning so they should be dragged close together; please consider only the aspect of ‘semantic similarity’ and disregard other aspects such as object size, color, materials, or pure associations (e.g., dog–bone).” The instruction was adapted from classical behavioral studies using semantic similarity ratings (e.g., [56]). To optimally estimate the dissimilarity matrix, all 100 images were only shown together in the first trial, and a subset was selected in every subsequent trial (see [27] for details). Modality-specific attribute (shape, manipulation, color, and motion) RDMs were based on the same arrangement method using different instructions (e.g., “Please arrange these objects according to their color/shape/manipulation/motion similarity”). Because some attributes may not be salient for some categories (e.g., it is not sensible to ask for the manipulation of a tiger or the motion of a monument), only those categories with explicit and lucid attributes were selected for a given attribute (i.e., shape for all five categories, small non-tool artifacts and tools for manipulation, fruits/vegetables and animals for color, and tools and animals for motion). All modality-specific attribute RDMs were then mapped to a complete 100 × 100 matrix by setting missing values to 1 (i.e., items without certain type of salient properties were labeled as being most dissimilar with other items on this property type; we also carried out an analysis, setting such missing values to NaN and the result pattern remained largely unchanged, see Table 1). Confounding variable RDMs were constructed based on the visual, phonological, and category properties of the items. We computed the low-level visual RDMs based on image silhouettes, because this method offers an effective prediction of the activation patterns in the early visual cortex [25]. The image pixels were binarized according to whether the pixel belonged to the object (pixel value = 1) or to the background (pixel value = 0). The dissimilarity between images was computed by 1 minus Jaccard similarity. For the phonological RDM, the dissimilarity of two-item names was measured by 1 minus the proportion of shared sub-syllabic units (onset or rhyme), regardless of position (e.g., [57]). The sub-syllabic units for a given syllable were defined based on the phonetic transcript of Chinese characters (the “pinyin” system), which transcribes each syllable with an onset consonant (“shengmu”) and a rhyme vowel or vowel-consonant (“yunmu”). The categorical RDM was constructed based on five object categories, with item pairs within the same category labeled 0 and other cells labeled 1.

### Neural RDM construction

We used structural-property-pattern (lesion)-based RSA to investigate semantic and modality-specific attribute representation in WM connections and GM regions. Similar to the conventional RSA, which is a highly fruitful method to research the neural representation in cortical regions using functional imaging data, the structural-property-pattern (lesion)-based RSA computes the relationship between the neural RDMs and behavioral or theoretical RDMs. The main difference is that the neural RDMs in this study were constructed by machine-learning models based on performances on neuropsychological tests and patients’ brain structural lesion patterns. The main rationale for this neural similarity measure is that if a WM connection pattern contains certain aspects of information shared by the naming process of two items (e.g., some semantic features), models trained with one item should also be able to predict naming accuracy of the other item to some degree. We first extracted the lesion features, balanced item labels by bootstrapping, input the lesion features and balanced labels into SVM training and testing to obtain the neural RDM, and used permutation to estimate the significance level of the neural RDM. The full pipeline is shown in Fig 1 and the details for each of these steps are described below in turn. The scripts of the full pipeline can be found at https://osf.io/h7upk/?view_only=52b8f86cffa14ed4844e4a1b9cd429cb.

#### Extracting the lesion features

As shown in Fig 1B, we first obtained the lesion mask (manually traced in T1 image) for each patient, then converted to MNI space, which was then overlapped with a WM connection template constructed from a healthy population [27], to extract the voxel-wise lesion pattern for each patient on each WM connection. We here focused on the structural (lesion) imaging data instead of performing analyses directly on patients’ DTI data (e.g., analyzing fractional anisotropy [FA] values or performing tractography), mainly because lesions from structural imaging (T1 and T2) are most straightforward in capturing brain structure damage properties in our specific patient type (mostly chronic stroke).

For lesion identification, in each patient, a lesion mask was constructed from manually traced lesion contours on averaged T1 images slice-by-slice with reference to T2 images (see [27] for details). Lesion mapping in patients with brain damage is a challenging task and various automatic methods have been developed, with supervised or nonsupervised algorithms [58–61], but manual drawing is considered the gold standard [58,62], even in very recent works [61]. We chose this highly labor-intensive method to ensure the validity of the lesion data and have gone through several procedures to ensure the reliability (inter-rater reliability values between our two investigators and an experienced radiologist were: mean percentage volume difference, 9% ± 8% and 4% ± 3%; mean percentage of discrepant voxels, 7% ± 4% and 6% ± 2%).

For WM connection, we adopted a previously reported template of the whole-brain WM network [27] to have a common reference template for the WM lesion patterns in the individual patients. Building neural RDMs in the current approach can only be done in the common template space where voxels are lined up, so that lesion patterns for different patients can be compared (i.e., for a same voxel, whether patients have lesion or not) and to be used as features for machine-learning model computation. The template we adopted was constructed using deterministic fiber tracking based on diffusion imaging data of 48 healthy participants ([27]; S1A Fig). This template contains 688 WM connections across 90 GM nodes (parcellated by the AAL atlas [63]). Briefly, the WM reconstruction was first applied in each healthy subject using determinative tracking among every two AAL regions. The resulting tracking maps in the subjects’ native space were transformed to a binary map in the MNI space. The binary maps of the MNI space for all subjects were then overlaid to generate a count map. Finally, a group-level threshold was set at voxel value (>25% of subjects; cluster size > 300 voxels) to determine whether a pair of brain regions was anatomically connected. The details of template construction can be found in [27]. Deterministic tracking was used because it has determinate termination conditions (FA values and fiber angles). It tends to suffer more false negatives but offers a clear border of WM connection to avoid invading to GM. While probabilistic tracking is generally considered more sensitive than deterministic tracking and thus revealing of more WM structures [64–66], it also increases the probability of false connections, and the biological meanings of the probabilistic values are uncertain, while it is relatively clear for the measurements used in deterministic tracking [64]. Note that the DTI imaging acquisition was suboptimal, according to standards nowadays, because of pragmatic issues in collecting patient and healthy group data using the same scanner. Nonetheless, the existence and shape of the connections showed generally good correspondence with WM networks constructed from other datasets (e.g., [67]; see also below for validation analyses).

Then, the patients’ lesion mask was converted to MNI space and overlapped with the WM connection template or the GM region masks (see [27] for details). In each WM connection/GM region, intact voxels (i.e., without lesion) were labeled 0 and lesioned voxels were labeled 1. This resulted in a binary V × N matrix in which V denoted the total number of voxels in the WM connection/GM region and N the number of patients, constituting a feature set for each machine-learning model. To ensure that the WM connections/GM regions had enough subjects with lesion coverage, we only tested the WM connections and GM regions with at least five subjects having damage and with more than 20 voxels lesioned per patient (see lesion distribution at S1E Fig). A total of 680 out of 688 connections and 80 out of 90 AAL regions were included in the following analyses. Because the input feature data only contained binary values and the range was consistent with the behavioral data, no normalization was applied in the feature set.

#### Bootstrapping

An item’s naming accuracy across all patients was not always 50%. Unbalanced training labels (e.g., the numbers of 0’s and 1’s in the training data were not equal) would ruin the classification ability because the training model always classifies the test sample into the group whose labels are predominant in number. A bootstrapping method was used to address this issue. Before classification, the subjects were reallocated into two groups: one group with correct responses and the other group with incorrect responses. We selected all subjects with the less common response of the two groups (e.g., if the accuracy of one item was 60%, all subjects with incorrect responses were selected) along with the same number of subjects randomly chosen from the other group. Thus, a new dataset for each item was constructed, with an accuracy across patients of 50%. The sample sizes of the training data for the 100 items ranged from 12 to 78 subjects (mean = 41.8 ± 8.5). This procedure was repeated 100 times for each item in each WM connection/GM region.

#### SVM training and testing

For each WM connection or GM region, a linear SVM with default parameters [68] was used. For each item (e.g., scissors), an SVM classifier was trained based on the balanced naming labels and voxel-wise lesion patterns. The resultant classifier was used to predict the naming score (1 or 0) of all patients who were not included in the training set using their lesion patterns; for patients who were included in the training set, a leave-two-out cross-validation scheme was used. This combined procedure ended up with a predicted score for each patient (each patient was a testing case once across testing iterations). The correspondence (simple matching coefficient) between this predicted score (based on training model of one item) and the actual naming score of each of the other items (e.g., axe) was calculated and was considered the neural similarity between the training item and this other item (i.e., scissors–axe similarity) on the particular WM connection being tested. All cross-item and within-item similarity could be obtained this way, resulting in a 100 × 100 similarity matrix. We averaged the symmetrical cells in the matrix according to the principal diagonal to obtain a symmetric matrix. Each cell in the matrix was then averaged across all 100 bootstrapped samples to produce the final 100 × 100 (1-similarity) neural RDM.

#### Significance testing (permutation and FDR)

The nonparametric permutation test (10,000 times) was used to estimate the significance of the classification model for each individual edge. For each permutation, the patient labels were randomly exchanged to shuffle the relationship between behavioral data and lesion data. The averaged accuracy of the principal diagonal cells (i.e., within-item prediction accuracies) was then computed. The *p*-value was calculated as the fraction of accuracies from all permutations that were greater than the actual accuracy using correct labels. For each WM connection, an independent classification model was built. To control for false positives caused by comparisons across multiple edges, we applied FDR as a multi-comparison correction method. The neural RDMs of WM connections/GM regions with significant within-item prediction accuracies at the threshold of FDR *q* < 0.05 were considered meaningful and were used for further analyses.

### Representational similarity analyses: Correlating neural RDMs with behavioral RDMs

The neural RDMs were correlated with behavioral RDMs using Spearman correlation. Specifically, for each WM connection, its neural RDM (a 100 [-item] × 100 [-item] matrix) and the semantic RDM (a 100 [-item] × 100 [-item] matrix) were both converted to a 1 × 4,950 vector. Correlation was computed on these two vectors (4,950 pairs of values). The *r* values were used to determine the extent of specific information encoded in the WM connections/GM regions. The FDR (*q* < 0.05) was used for multiple comparison correction. To investigate the higher-order semantic effects beyond modality-specific attributes, partial correlation analyses were performed between the semantic RDM and neural RDMs, with the modality-specific attribute RDMs (and the peripheral and categorical matrices) as nuisance variables. As explained in the “Behavioral RDM Construction” session, we adopted two ways of treating missing values in the modality-specific attributes (e.g., animal items were not rated on “manipulation” property)—setting it to be 1 (most dissimilar with other items on this modality) or to “NaN” (missing value). The RSA mapping procedure was implemented using a custom MATLAB function.

### Validation analyses

#### Quality of the WM fiber tracking

We used the HCP database to check the WM template that we used in the main analyses, because HCP contains high-quality diffusion MRI data with advanced acquisition and processing methods [69]. Diffusion scans were acquired in a Siemens 3T Skyra scanner using a 2D spin-echo single-shot multiband EPI sequence with a multiband factor of three and a monopolar gradient pulse. The spatial resolution was 1.25 mm isotropic, TR = 5,500 ms, TE = 89 ms. A multishell diffusion scheme was used. The b-values were 1,000, 2,000, and 3,000 s/mm^2^. The total number of diffusion sampling directions was 270. We used the dataset “unrelated 40” on the ConnectomeDB website (https://db.humanconnectome.org/) for fiber reconstruction. After excluding two subjects with technical problems in acquisition, the remaining 38 subjects were included in the analyses. The preprocessing, reconstruction, and fiber tracking were performed with DSI-studio software (dsi-studio.labsolver.org). To reduce the fiber-crossing problem, we reconstructed the diffusion data using the generalized q-sampling imaging (GQI) method [70].

#### Controlling for the effects of patient disease type and lesion hemisphere

We computed the neural RDMs in WM connections using data from the 67 stroke patients or from the 30 patients with unilateral left hemispheric lesions.

## Supporting information

S1 Fig The WM template and the results of lesion-naming predictions. (A) The WM template used in the current study was adopted from Fang et al. (2015), in which deterministic tractography was performed across 90 AAL regions using the DTI data of 48 healthy adults acquired in the same scanner as our patient imaging data. The resulting whole-brain anatomical network contained 688 WM connections. (B) Patients’ naming performance distribution for the 100 objects. (C) The 197 WM connections in which the lesion pattern predicted the naming performance for the same items with greater-than-chance accuracy in the SVM model. The RSA analyses were conducted on these connections. (D) The 60 WM connections in which the neural RDMs significantly positively correlated with the semantic RDM before controlling for the attribute RDMs (FDR *q* < 0.05). (E) Patient’s lesion distribution in the WM connections and GM nodes. The *N* value of each WM connection and each GM node was denoted by the number of patients with lesion in more than 20 voxels. The brain figures were generated using Brainnet Viewer (Xia et al. 2013). AAL, automated anatomical labeling; DTI, diffusion tensor imaging; FDR, false discovery rate; GM, gray matter; RDM, representational dissimilarity matrix; RSA, representational similarity analysis; SVM, support vector machine; WM, white matter.
(DOCX)

S2 Fig Reconstruction of the eight WM connections that represent higher-order semantic space. The masks of the WM connections that were used in our main analyses (adopted from Fang et al. 2015) are shown in the two left columns, and the WM connections reconstructed using Human Connectome Project data are shown in the two right columns. The brain figures were generated using BrainNet Viewer (Xia et al. 2013). WM, white matter.
(DOCX)

S1 Table The RSA results in the GM nodes that are connected by the WM connections that showed robust higher-order semantic effect. ^*^Positive correlation values that survived FDR correction (*q* < 0.05). The missing values were set as “1” (most dissimilar) in modality-specific attributes matrix. ^#^Low-level visual, phonological, category. FDR, false discovery rate; GM, gray matter; MTG, middle temporal gyrus; MidATL, middle anterior temporal lobe; RSA, representational similarity analysis; SupATL, superior anterior temporal lobe; STG, superior temporal gyrus; WM, white matter.
(DOCX)

S2 Table Background information of the 80 patients. (DOCX)

## Abbreviations

AAL: automated anatomical labeling
ATL: anterior temporal lobe
DTI: diffusion tensor imaging
FA: fractional anisotropy
FDR: false discovery rate
fMRI: functional magnetic resonance imaging
FOV: field of view
GM: gray matter
GQI: generalized q-sampling imaging
HARDI: high angular resolution diffusion imaging
HCP: Human Connectome Project
IFOF: inferior fronto-occipital fasciculus
ILF: inferior longitudinal fasciculus
IPL: inferior parietal lobule
MTG: middle temporal gyrus
NaN: not a number
RDM: representational dissimilarity matrix
RSA: representational similarity analysis
STG: superior temporal gyrus
SVM: support vector machine
TBI: traumatic brain injury
TE: echo time
TR: repetition time
UF: uncinate fasciculus
WM: white matter
