# Supplementary material for: Semantic representation in the white matter pathway
Source: PLoS Biol. 2018 Apr 6;16(4):e2003993. doi: 10.1371/journal.pbio.2003993 (PMC5906027; doi:10.1371/journal.pbio.2003993)
Supplement: S1 Fig — (A) The WM template used in the current study was adopted from Fang et al. (2015), in which deterministic tractography was performed across 90 AAL regions using the DTI data of 48 healthy adults acquired in the same scanner as our patient imaging data. The resulting whole-brain anatomical network contained 688 WM connections. (B) Patients’ naming performance distribution for the 100 objects. (C) The 197 WM connections in which the lesion pattern predicted the naming performance for the same items with greater-than-chance accuracy in the SVM model. The RSA analyses were conducted on these connections. (D) The 60 WM connections in which the neural RDMs significantly positively correlated with the semantic RDM before controlling for the attribute RDMs (FDR q < 0.05). (E) Patient’s lesion distribution in the WM connections and GM nodes. The N value of each WM connection and each GM node was denoted by the number of patients with lesion in more than 20 voxels. The brain figures were generated using Brainnet Viewer (Xia et al. 2013). AAL, automated anatomical labeling; DTI, diffusion tensor imaging; FDR, false discovery rate; GM, gray matter; RDM, representational dissimilarity matrix; RSA, representational similarity analysis; SVM, support vector machine; WM, white matter. (DOCX) [file pbio.2003993.s001.docx]

**
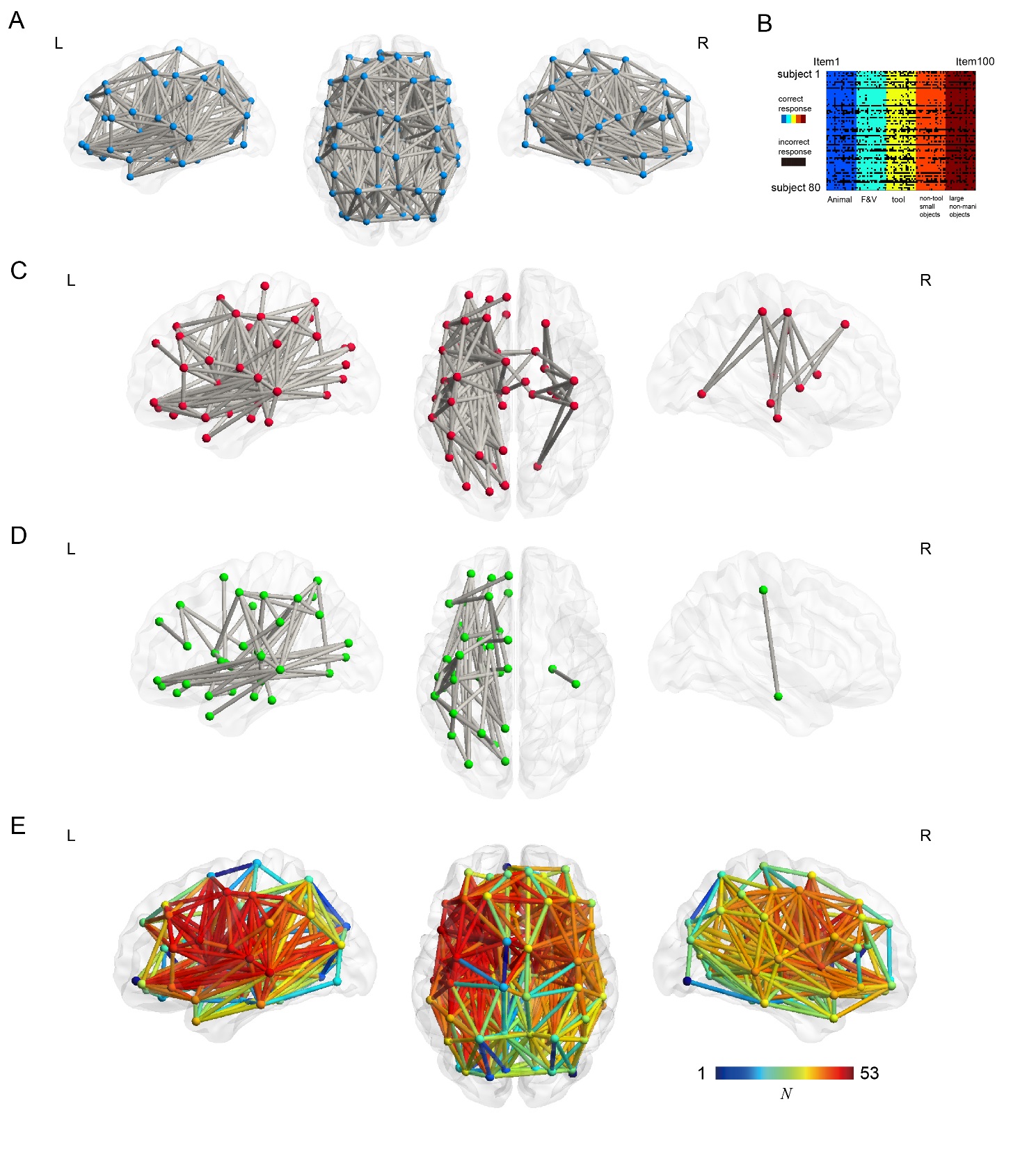
 S1 Fig**. **The WM template and the results of lesion-naming predictions.**

(A) The WM template used in the current study was adopted from Fang et al. (2015), where deterministic tractography was performed across 90 AAL regions using the DTI data of 48 healthy adults acquired in the same scanner as our patient imaging data. The resulting whole-brain anatomical network contained 688 WM connections. (B) Patient’s naming performance distribution for the 100 objects. (C) The 197 WM connections in which the lesion pattern predicted the naming performance for the same items with greater-than-chance accuracy in the SVM model. The RSA analyses were conducted on these connections. (D) The 60 WM connections in which the neural RDMs significantly positively correlated with the semantic RDM before controlling for the attribute RDMs (FDR *qs* < 0.05). (E) Patient’s lesion distribution in the WM connections and GM nodes. The *N* value of each WM connection and each GM node was denoted by the number of patients with lesion in more than 20 voxels. The brain figures were generated using Brainnet Viewer (Xia et al., 2013).
