# Supplementary material for: Semantic representation in the white matter pathway
Source: PLoS Biol. 2018 Apr 6;16(4):e2003993. doi: 10.1371/journal.pbio.2003993 (PMC5906027; doi:10.1371/journal.pbio.2003993)
Supplement: S2 Fig — The masks of the WM connections that were used in our main analyses (adopted from Fang et al. 2015) are shown in the two left columns, and the WM connections reconstructed using Human Connectome Project data are shown in the two right columns. The brain figures were generated using BrainNet Viewer (Xia et al. 2013). WM, white matter. (DOCX) [file pbio.2003993.s002.docx]

**
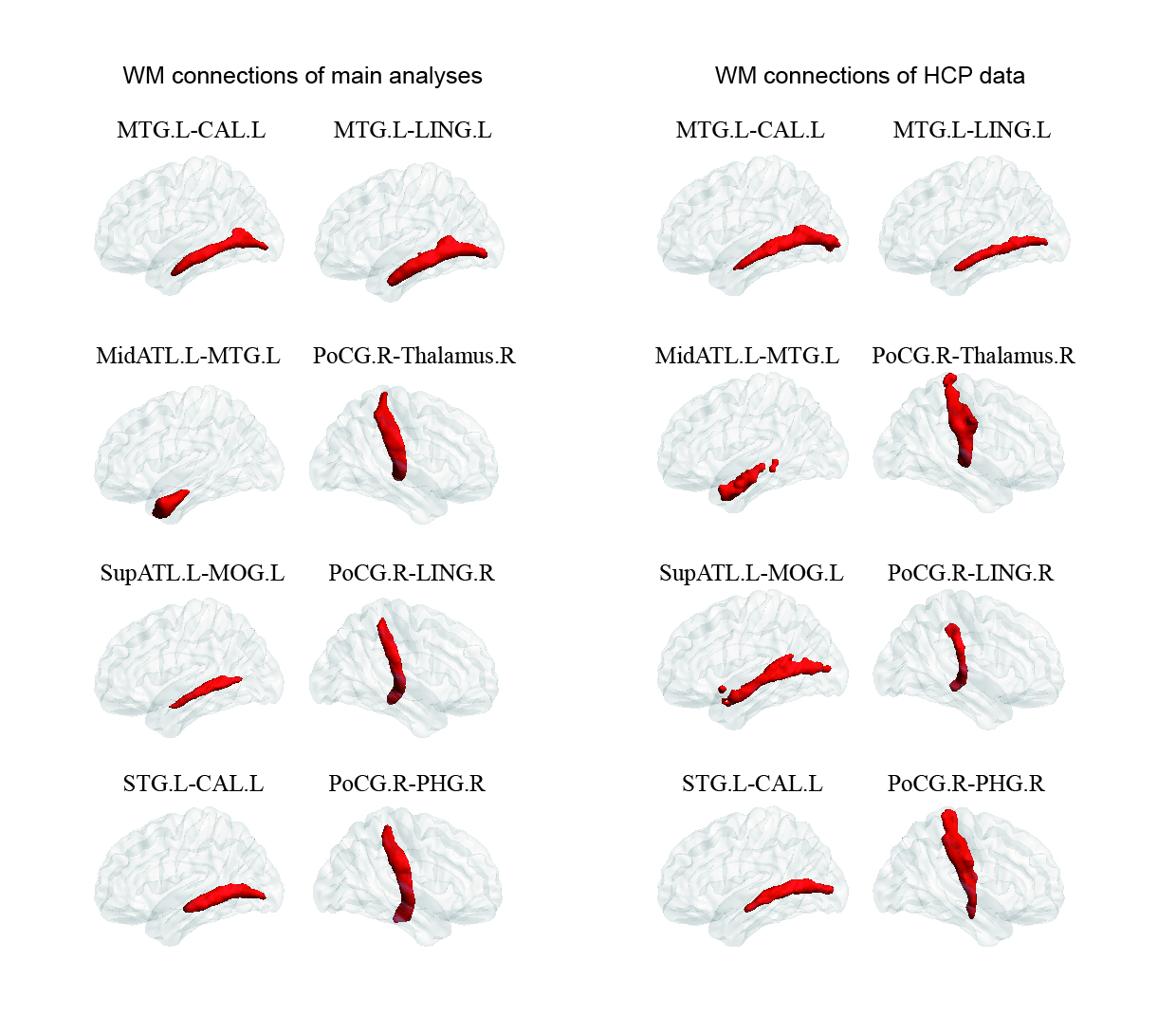
**

**S2 Fig.** **Reconstruction of the eight WM connections that represent higher-order semantic space.**

The masks of the WM connections that were used in our main analyses (adopted from Fang et al. 2015) are shown in the two left columns, and the WM connections reconstructed using Human Connectome Project data are shown in the two right columns. The brain figures were generated using Brainnet Viewer (Xia et al., 2013).
