# Supplementary material for: Semantic representation in the white matter pathway
Source: PLoS Biol. 2018 Apr 6;16(4):e2003993. doi: 10.1371/journal.pbio.2003993 (PMC5906027; doi:10.1371/journal.pbio.2003993)
Supplement: S1 Table — *Positive correlation values that survived FDR correction (q < 0.05). The missing values were set as “1” (most dissimilar) in modality-specific attributes matrix. #Low-level visual, phonological, category. FDR, false discovery rate; GM, gray matter; MTG, middle temporal gyrus; MidATL, middle anterior temporal lobe; RSA, representational similarity analysis; SupATL, superior anterior temporal lobe; STG, superior temporal gyrus; WM, white matter. (DOCX) [file pbio.2003993.s003.docx]

The RSA results in the GM nodes that are connected by the WM connections that showed robust higher-order semantic effect. *: positive correlation values that survived FDR correction (*q* < 0.05). The missing values were setting as “1” (most dissimilar) in modality-specific attributes matrix. #: low-level visual, phonological, category. Abbreviation: MTG, middle temporal gyrus; MidATL, middle anterior temporal lobe; SupATL, superior anterior temporal lobe; STG, superior temporal gyrus.

| **Analysis aspect** | **Control variables** | **GM node being connected with the connections which significant in higher-order semantic RSA** | | | |
| --- | --- | --- | --- | --- | --- |
|  |  | **STG** | **SupATL** | **MTG** | **MidATL** |
| **Higher-order semantic RSA** | |  |  |  |  |
|  | *(control for modality-specific attributes and the other relevant matrices^#^)* | | | | |
|  |  | -0.02 | -0.015 | -0.045 | -0.023 |
| **Semantic RSA** |  |  |  |  |  |
|  | *(without controlling for modality-specific attributes, and control for the other relevant matrices^#^)* | | | | |
|  |  | 0.025(0.077) | 0.036(0.012*) | 0.013(0.346) | -0.009 |
| **Shape attribute RSA** | |  |  |  |  |
|  | *(control for the other relevant matrices^#^)* | | | |  |
|  |  | 0.065(<0.001*) | 0.044(0.002*) | 0.078(<1×10^-6^*) | 0.037(0.01*) |
| **Manipulation attribute RSA** | |  |  |  |  |
|  | *(control for the other relevant matrices^#^)* | | | |  |
|  |  | 0.12(<1×10^-6^*) | 0.119(<1×10^-6^*) | 0.163(<1×10^-6^*) | -0.01 |
| **Color attribute RSA** | |  |  |  |  |
|  | *(control for the other relevant matrices^#^)* | | | |  |
|  |  | -0.039 | 0.001(0.94) | -0.071 | 0.062(<0.001*) |
| **Motion attribute RSA** | |  |  |  |  |
|  | *(control for the other relevant matrices^#^)* | | | |  |
|  |  | -0.072 | -0.045 | -0.099 | -0.056 |
