# Supplementary material for: Semantic representation in the white matter pathway
Source: PLoS Biol. 2018 Apr 6;16(4):e2003993. doi: 10.1371/journal.pbio.2003993 (PMC5906027; doi:10.1371/journal.pbio.2003993)
Supplement: S2 Table — (DOCX) [file pbio.2003993.s004.docx]

Background information of the 80 patients

|  |
| --- |

| **Code** | **Sex** | **Age (year)** | **Education (year)** | **Post-onset of disease**  **(month)** | **Cause of disease** | **Lesion site** | | | | | **Lesion percentage** | | |
| --- | --- | --- | --- | --- | --- | --- | --- | --- | --- | --- | --- | --- | --- |
|  |  |  |  |  |  | **Left hemisphere** | | **Right hemisphere** | | **Brain stem** | **In GM** | **In WM** | **The WM part overlapped with common WM mask** |
|  |  |  |  |  |  | **GM** | **WM** | **GM** | **WM** |  |  |  |  |
| 4 | male | 49 | 16 | 1 | hemorrhage | F, P, T, Ins, BG | * | - | - | - | 38.35% | 51.82% | 100.00% |
| 6 | female | 35 | 12 | 21 | infarction | F, P, T, Lim, Ins, BG, Thal | * | - | - | - | 45.11% | 31.14% | 99.94% |
| 7 | male | 48 | 15 | 3 | infarction | Lim, BG | * | - | * | - | 13.20% | 80.24% | 100.00% |
| 8 | male | 60 | 16 | 1 | hemorrhage | F, P, T, Lim, Ins, BG | * | F, Lim | * | - | 37.61% | 36.53% | 100.00% |
| 9 | male | 43 | 19 | 10 | infarction | - | - | Lim, BG | * | - | 3.00% | 96.80% | 100.00% |
| 10 | male | 51 | 15 | 6 | cerebrovascular disorder | P, O, T, Ins | * | - | - | - | 50.90% | 33.27% | 99.96% |
| 15 | male | 22 | 12 | 86 | trauma | P, O, Lim | * | - | - | - | 41.68% | 34.84% | 99.96% |
| 16 | male | 32 | 15 | 8 | hemorrhage | F, O, T, Lim, Ins, BG | * | - | - | - | 54.97% | 36.60% | 100.00% |
| 18 | male | 34 | 12 | 8 | hemorrhage | F, P, T, Lim, Ins, BG | * | - | - | - | 48.17% | 32.45% | 100.00% |
| 19 | male | 42 | 14 | 5 | infarction | F, P, T, Lim, Ins, BG | * | - | - | - | 46.46% | 35.56% | 99.95% |
| 21 | male | 63 | 12 | 1 | infarction | - | - | P, Ins, Lim, Thal | * | - | 21.66% | 78.03% | 100.00% |
| 22 | male | 49 | 12 | 2 | infarction | F, P, T, Ins, Lim, BG, Thal | * | - | - | - | 46.65% | 35.96% | 100.00% |
| 25 | male | 46 | 16 | 1 | infarction | - | - | O, T, BG, Thal | * | - | 38.26% | 60.67% | 100.00% |
| 29 | male | 30 | 19 | 2 | infarction | - | - | F, P, T, Lim, Ins, BG, Thal | * | * | 42.55% | 43.87% | 100.00% |
| 30 | male | 41 | 15 | 7 | hemorrhage | T, Ins, Lim, BG, Thal | * | - | - | - | 29.99% | 69.54% | 100.00% |
| 33 | male | 36 | 15 | 9 | hemorrhage | F, Ins, BG, Thal | * | - | - | - | 25.35% | 72.20% | 100.00% |
| 34 | female | 64 | 12 | 4 | infarction | F, Ins, BG, Thal | * | - | - | - | 9.97% | 88.01% | 100.00% |
| 35 | male | 46 | 12 | 5 | infarction | BG | * | BG, Thal | * | - | 13.93% | 83.02% | 100.00% |
| 38 | male | 40 | 12 | 2 | hemorrhage | - | - | F, T, Ins, Lim, Thal | * | - | 22.45% | 74.82% | 100.00% |
| 48 | female | 56 | 12 | 5 | hemorrhage | - | - | F, Ins. BG, Thal | * | * | 17.34% | 82.43% | 100.00% |
| 56 | male | 61 | 15 | 1 | hemorrhage | F | * | F, O, Ins, BG | * | - | 24.67% | 72.38% | 100.00% |
| 57 | male | 51 | 9 | 1 | infarction | F, P, T, Ins, Lim | * | - | - | - | 40.50% | 47.50% | 99.96% |
| 60 | male | 45 | 16 | 3 | infarction | T, Lim, Ins, BG, Thal | * | - | - | * | 29.41% | 65.13% | 100.00% |
| 62 | female | 56 | 12 | 5 | infarction | F, P, O, T, Ins, BG | * | - | - | - | 31.66% | 66.62% | 100.00% |
| 66 | male | 35 | 16 | 1 | infarction | - | - | F, Lim, BG, Ins, Thal | * | - | 29.02% | 66.74% | 100.00% |
| 67 | male | 47 | 9 | 4 | infarction | - | - | F, P, Ins, Lim, BG, Thal | * | * | 22.65% | 74.32% | 100.00% |
| 69 | female | 76 | 2 | 4 | thrombosis | F, P, T, Ins, Lim, BG, Thal | * | - | * | * | 22.01% | 74.92% | 100.00% |
| 72 | male | 41 | 9 | 1 | hemorrhage | F, T, Ins, BG | * | - | - | - | 33.21% | 62.32% | 100.00% |
| 78 | male | 40 | 8 | 1 | trauma | T | * | F, T | * | - | 37.27% | 11.92% | 100.00% |
| 79 | male | 48 | 19 | 23 | hemorrhage | - | * | P, F, O, T, Lim, Ins, BG, Thal | * | * | 43.92% | 44.00% | 99.94% |
| 82 | female | 35 | 15 | 16 | hemorrhage | P, F, Ins, BG, Thal | * | - | - | - | 32.97% | 62.40% | 99.89% |
| 85 | male | 67 | 9 | 6 | infarction | F, P, T, Ins, Lim, BG | * | - | - | - | 40.82% | 38.95% | 100.00% |
| 86 | male | 67 | 12 | 4 | thrombosis | F, Lim, Ins, BG | * | F, Lim, BG | * | - | 11.17% | 88.17% | 100.00% |
| 87 | female | 45 | 9 | 30 | trauma | BG, Thal | * | F, P, T, Lim, Ins, BG | * | * | 46.49% | 32.39% | 100.00% |
| 88 | female | 70 | 16 | 2 | trauma | F, P, T, Lim, Ins | * | F, BG, L,Ins | * | - | 34.55% | 46.47% | 100.00% |
| 89 | male | 46 | 9 | 1 | infarction | F, P, T, Lim, BG | * | F, P, Lim, BG | * | - | 36.39% | 54.27% | 99.97% |
| 91 | male | 65 | 9 | 8 | infarction | F, P, T, Lim, BG, Thal | * | F, P, O, T, Lim, Ins, BG | * | * | 15.36% | 82.87% | 100.00% |
| 97 | male | 55 | 15 | 2 | infarction | F, Ins, BG | * | F, P, T, Lim, BG, Thal | * | * | 37.62% | 50.23% | 100.00% |
| 98 | male | 62 | 12 | 9 | infarction | Ins, Lim, BG | * | F, P, Lim, BG | * | - | 9.83% | 89.09% | 100.00% |
| 101 | male | 35 | 16 | 2 | infarction | F, P, T, Lim, BG | * | - | - | - | 45.30% | 37.92% | 100.00% |
| 102 | male | 28 | 16 | 2 | infarction | F, P, O, T, Ins, BG | * | - | - | - | 49.25% | 37.03% | 100.00% |
| 103 | female | 37 | 12 | 2 | infarction | F, P, O, T | * | - | - | - | 38.96% | 43.26% | 99.92% |
| 106 | female | 22 | 16 | 1 | hemorrhage | - | * | F, P, Lim, BG | * | - | 24.53% | 73.18% | 100.00% |
| 109 | male | 58 | 9 | 7 | infarction | F, P, Lim, Ins, BG | * | BG | * | - | 27.14% | 70.50% | 100.00% |
| 111 | female | 51 | 8 | 4 | infarction | Ins, BG | * | F, P, O, T, Lim, Ins, BG | * | - | 41.81% | 50.90% | 99.94% |
| 112 | male | 22 | 15 | 10 | trauma | T, Ins, BG | * | - | - | - | 31.18% | 27.56% | 100.00% |
| 115 | male | 20 | 9 | 1 | hemorrhage | F, O, T | * | F, T | * | - | 38.23% | 15.51% | 100.00% |
| 116 | male | 56 | 15 | 4 | infarction | F, P, T, Lim, Thal | * | - | - | * | 37.14% | 47.06% | 100.00% |
| 117 | female | 45 | 15 | 1 | infarction | - | - | F, P, O, T, Lim, Ins, BG | * | - | 43.92% | 42.50% | 99.99% |
| 118 | female | 40 | 12 | 7 | infarction | P, F, T, Ins, BG, Thal | * | - | - | - | 42.11% | 39.82% | 99.99% |
| 123 | male | 24 | 9 | 1 | trauma | - | - | P, F, Lim, Ins | * | - | 29.59% | 53.28% | 99.75% |
| 125 | male | 48 | 9 | 2 | hemorrhage | - | * | P, F, O, T, Lim, Ins, BG | * | * | 40.11% | 46.09% | 100.00% |
| 129 | male | 58 | 15 | 1 | infarction | F, P, Lim, Ins, BG | * | - | - | - | 38.47% | 52.57% | 100.00% |
| 132 | male | 37 | 12 | 2 | infarction | F, P, O, T, Ins, Lim, BG, Thal | * | - | * | - | 9.44% | 90.39% | 100.00% |
| 133 | male | 47 | 15 | 2 | infarction | F, P, T, Lim, Ins, BG, Thal | * | - | - | * | 37.48% | 56.72% | 100.00% |
| 159 | female | 39 | 15 | 2 | hemorrhage | - | - | T, Lim, Ins, BG, Thal | * | - | 35.78% | 57.18% | 99.99% |
| 161 | male | 53 | 12 | 1 | infarction | Lim, P, Thal | * | F, P, O, T, Lim, Ins, BG | * | - | 24.13% | 74.05% | 100.00% |
| 162 | male | 48 | 15 | 2 | hemorrhage | F, P, O, T, Ins, BG | * | - | * | - | 6.05% | 93.62% | 100.00% |
| 163 | male | 47 | 16 | 1 | infarction | F, BG, Ins | * | BG | * | - | 19.31% | 77.43% | 100.00% |
| 165 | male | 47 | 16 | 4 | hemorrhage | F, P, T, Ins, BG | * | F, BG | * | - | 25.21% | 71.90% | 100.00% |
| 175 | male | 20 | 15 | 1 | trauma | P, T | * | - | - | - | 46.20% | 39.43% | 99.99% |
| 177 | male | 26 | 16 | 2 | infarction | F, P, T, Lim, Ins, Thal | * | - | - | - | 46.57% | 46.92% | 100.00% |
| 180 | female | 41 | 15 | 4 | hemorrhage | F, P, T, Ins, BG | * | - | - | - | 26.11% | 70.28% | 100.00% |
| 181 | male | 49 | 15 | 4 | hemorrhage | BG, Ins | * | F, P, T, Lim, BG, Thal | * | * | 12.35% | 86.59% | 100.00% |
| 182 | female | 40 | 15 | 2 | infarction | F, P, O, T, BG, Thal | * | F, P, Lim, T, Ins, BG | * | - | 11.11% | 87.09% | 100.00% |
| 185 | male | 57 | 6 | 1 | infarction | - | - | Ins, BG, Thal | * | - | 16.78% | 83.14% | 100.00% |
| 190 | male | 42 | 12 | 5 | hemorrhage | Lim, BG, Thal | * | - | - | - | 17.92% | 81.74% | 100.00% |
| 192 | male | 21 | 12 | 46 | trauma | Ins, BG | * | F, BG | * | * | 12.30% | 87.54% | 100.00% |
| 196 | male | 56 | 2 | 1 | infarction | P, F, O, T, BG | * | Lim, BG |  | - | 6.74% | 93.03% | 100.00% |
| 205 | male | 47 | 12 | 1 | trauma | - | - | BG, Ins, Thal | * | - | 9.70% | 88.74% | 100.00% |
| 206 | male | 40 | 15 | 1 | hemorrhage | T, Ins, BG, Thal | * | - | - | * | 34.83% | 57.47% | 100.00% |
| 208 | male | 45 | 9 | 1 | trauma | - | - | P, T | - | - | 39.62% | 59.16% | 100.00% |
| 211 | female | 52 | 6 | 2 | infarction | - | - | P, F, T, Lim, BG | * | - | 32.82% | 58.71% | 100.00% |
| 213 | male | 55 | 15 | 1 | hemorrhage | P, T, O, Ins | * | - | - | - | 43.49% | 49.17% | 100.00% |
| 214 | female | 19 | 12 | 1 | trauma | P, O, T | * | - | - | - | 49.03% | 19.76% | 100.00% |
| 218 | female | 25 | 12 | 8 | trauma | - | - | P, F, O, T, Ins, BG | * | * | 45.76% | 45.60% | 99.94% |
| 222 | female | 37 | 9 | 6 | trauma | BG | - | - | - | - | 0.75% | 99.18% | 100.00% |
| 231 | male | 43 | 15 | 3 | infarction | F, Ins | - | - | - | * | 22.12% | 77.79% | 100.00% |
| 304 | male | 70 | 12 | 1 | infarction | F, P, O, T, Lim, Ins, BG | * | F, BG | * | - | 41.50% | 46.03% | 100.00% |
| 305 | male | 47 | 12 | 3 | infarction | F, Ins, BG | * | - | - | - | 15.67% | 83.74% | 100.00% |

Note: LH: left hemisphere; RH: right hemisphere; F: frontal lobe; P: parietal lobe; O: occipital lobe; T: temporal lobe; Lim: limbic lobe; Ins: insula; BG: basal ganglia; Thal: thalamus; *: had lesion; -: Intact.
